# Out-patient commitment order use in Norway: incidence and prevalence rates, duration and use of mental health services from the Norwegian Outpatient Commitment Study

**DOI:** 10.1192/bjo.2019.60

**Published:** 2019-09-02

**Authors:** Henriette Riley, Ekaterina Sharashova, Jorun Rugkåsa, Olav Nyttingnes, Tore Buer Christensen, Ann-Torunn Andersen Austegard, Maria Løvsletten, Bjørn Lau, Georg Høyer

**Affiliations:** Research Director, Division of Mental Health and Substance Abuse, University Hospital of North Norway, Norway; Postdoctoral Fellow, Department of Community Medicine, UiT The Arctic University of Norway, Norway; Professor, Health Services Research Unit, Akershus University Hospital; and Centre for Care Research, University of South-Eastern Norway, Norway; Postdoctoral Fellow, Health Services Research Unit, Akershus University Hospital; and R&D Department, Division of Mental Health Services, Akershus University Hospital, Norway; Clinical Psychiatrist, Department of Mental Health, Sørlandet Hospital, Norway; Senior Advisor, Division of Psychiatry, Haukeland University Hospital, Norway; Doctoral Research Fellow, Division of Mental Health Care, Innlandet Hospital Trust, Norway; Professor, Lovisenberg Diaconal Hospital, Norway; Professor Emeritus, Division of Mental Health and Substance Abuse, University Hospital of North Norway; and Department of Community Medicine, UiT The Arctic University of Norway, Norway

**Keywords:** Outpatient commitment, community treatment order, involuntary out-patient treatment

## Abstract

**Background:**

Norway authorised out-patient commitment in 1961, but there is a lack of representative and complete data on the use of out-patient commitment orders.

**Aims:**

To establish the incidence and prevalence rates on the use of out-patient commitment in Norway, and how these vary across service areas. Further, to study variations in out-patient commitment across service areas, and use of in-patient services before and after implementation of out-patient commitment orders. Finally, to identify determinants for the duration of out-patient commitment orders and time to readmission.

**Method:**

Retrospective case register study based on medical files of all patients with an out-patient commitment order in 2008–2012 in six catchment areas in Norway, covering one-third of the Norwegian population aged 18 years or more. For a subsample of patients, we recorded use of in-patient care 3 years before and after their first-ever out-patient commitment.

**Results:**

Annual incidence varied between 20.7 and 28.4, and prevalence between 36.5 and 48.9, per 100 000 population aged 18 years or above. Rates differed significantly between catchment areas. Mean out-patient commitment duration was 727 days (s.d. = 889). Use of in-patient care decreased significantly in the 3 years after out-patient commitment compared with the 3 years before. Use of antipsychotic medication through the whole out-patient commitment period and fewer in-patient episodes in the 3 years before out-patient commitment predicted longer time to readmission.

**Conclusions:**

Mechanisms behind the pronounced variations in use of out-patient commitment between sites call for further studies. Use of in-patient care was significantly reduced in the 3 years after a first-ever out-patient commitment order was made.

**Declaration of interest:**

None.

The legal possibility of subjecting out-patients to mandatory care has steadily increased in recent decades, especially in North America, Oceania and Europe.^1–4^ Norway authorised out-patient commitment as early as in 1961, when patients discharged with an out-patient commitment order could be recalled to in-patient care without any further formalities. In 1999, the Norwegian Mental Health Act was amended, authorising out-patient commitment without a prior in-patient period. The coercive power of out-patient commitment orders in Norway is restricted to an obligation to comply with all scheduled treatment appointments, if necessary with the assistance of the police and the use of force. The legal criteria for out-patient commitment are the same as for in-patient commitment, including a requirement to make a global assessment as to whether the intervention is the best option for the patient or not. Out-patient commitment orders are valid for 1 year, but can be renewed by an independent review board an unlimited number of times. It is also noteworthy that the Norwegian Mental Health Act requires separate orders for involuntary placement and involuntary treatment, for both in- and out-patients. Norway is among the countries with the highest number of mental health workers per capita (240 per 100 000 population).^5^ The majority of these are in out-patient services, and there has been a drive to allocate an increasing proportion of resources to community services in recent years. Further details about the Norwegian out-patient commitment population, the Norwegian out-patient commitment framework and what being on an out-patient commitment entailed for patients is described in our previous paper from the Norwegian Outpatient Commitment Study (NOCS).^6^

Internationally, out-patient commitment has raised questions about the justification and effect of the order: does out-patient commitment reduce the overall use of coercion in the care of people with severe mental disorders, or does it increase coercion by adding coercive care in the community to in-patient coercion?^7,8^ Another frequently raised question concerns the impact of out-patient commitment on the use of mental health services, especially in-patient care.^4,9–11^ Further, perceived coercion of patients with out-patient commitment orders has received increased attention in recent years.^12,13^ The aim of NOCS was to collect representative and complete data on the use of out-patient commitment in Norway. In this paper, we report on population-based incidence and prevalence rates, and changes in the utilisation of mental health care services before and after patients were subjected to out-patient commitment, as well as predictors for the duration of out-patient commitment orders and time to readmission.

## Method

### Study design

NOCS is a retrospective case register study based on the examination of medical files of all patients with an out-patient commitment order between 2008 and 2012 in six Norwegian catchment areas. For the subsample of those who received their first-ever out-patient commitment order in 2008 and 2009, we recorded the use of in-patient services 3 years before and after the out-patient commitment order.

### Settings

The six study sites were located in different geographical areas across Norway. Sites were strategically selected in order to include both rural and urban populations. The areas are described elsewhere.^6^ The sites were the University Hospital of North Norway, Helse Bergen Trust, Sørlandet Hospital Trust, Innlandet Hospital Trust, Akershus University Hospital and Lovisenberg Hospital. The latter two are located in the Oslo area, the capital. The total population in the combined catchment areas equals one-third of the total Norwegian population aged 18 years or over, which was 3 867 645 in 2012.

### Participants

All patients aged 18 years or above who were on an out-patient commitment order at the beginning of 2008 were included in the study. Further, we added all new out-patient commitment orders made from 1 January 2008 to 31 December 2012. The total population in the combined catchment areas was 867 645 people (aged 18 years or above, 2012 figures). We identified a total of 1414 patients, representing 1700 out-patient commitment orders; of these, 274 patients received their first out-patient commitment in 2008 and 2009. Of all patients, 226 (16.0%) had two out-patient commitment episodes and 30 (2.1%) had three out-patient commitment episodes. More than half of the patients (52.8%) were in the 30- to 50-year age group, with a slight predominance of men (56.4%). Diagnoses were only recorded for a subsample of 274 patients, and showed that 76.9% were diagnosed within the schizophrenia disorders spectrum, 16.5% with affective mood disorders and 6.6% other diagnoses.

### Exclusion criteria

Participating hospitals sometimes appeared to use out-patient commitment to facilitate in-patient transfers from one service or ward to another, and many orders consequently ended after a few days. As we considered such very short term out-patient commitments not to relate to community treatment, we excluded these, and any other out-patient commitment orders lasting for fewer than 7 days. We also excluded involuntary in-patients placed on out-patient commitment during stays in general hospitals, as well as people who were placed on out-patient commitment following admission in one of the participating sites but who lived and received community care outside the catchment area. For those who moved out of the area or country, we censored data on the date they moved.

### Data collection and data management

Data were collected from the electronic medical records at each site between 1 January 2008 and 1 January 2012. To identify all patients who met the inclusion criteria, we searched the administrative electronic system (DIPS) used at all study sites. We cooperated closely with administrative staff working with this system to identify all potential participants. Data from patients' files were transferred to a registration form created specifically for the study. Five cases were piloted, which resulted in minor adjustments to the registration form. In a few cases, data were incongruent or incomplete. In such cases, we closely examined patients' entire records, including relevant referral notes, discharge notes, nurses' notes, reports from out-patient services, and correspondence between services, including out-patient clinics. All data were meticulously checked to resolve potential discrepancies in the data-set and we held regular team meetings.

For the total sample, we recorded a limited number of variables: demography, current out-patient commitment status, number and duration of out-patient commitment episodes in 2008–2012, and whether patients received an involuntary treatment order in conjunction with the out-patient commitment order or not. For those with their first-ever out-patient commitment order in 2008 and 2009 we recorded more comprehensive data (58 variables), including their consumption of in-patient care 3 years before and after the first-ever out-patient commitment order. The in-patient's admission resulting in the patient's first-ever out-patient commitment was included in the number of in-patient-episodes 3 years before out-patient commitment. Total numbers of involuntary in-patient placements each year from 2008 to 2013 were obtained through routine annual reports at each participating site.

### Statistical analyses

Incidence rates and point prevalence rates were calculated per 100 000 population (18 years of age or above) for each study year from 2008 to 2012. Poisson regression was used to test for overall difference in incidence and point prevalence rates between sites. To describe the sample and subsamples, we calculated means (standard deviations) and medians (first and third quartiles) for continuous variables, while proportions were calculated for categorical variables. The Mann–Whitney *U*-test, Kruskal-Wallis *H*-test, Wilcoxon signed-ranks test and Pearson's chi-squared test were used to compare groups when appropriate.

Kaplan–Meier survival analysis with log-rank tests was performed to estimate crude associations between duration of the first-ever out-patient commitment order and the following variables: site, gender, age, diagnosis, time since first contact with psychiatric services, use of psychiatric services before out-patient commitment, living alone or not, involuntary admissions before first-ever out-patient commitment order, reference to danger to self or others, involuntary depot medication and follow-up variables. The same analysis was performed for time to readmission. We used Cox regression analyses to estimate independent associations and to calculate mutually adjusted hazard ratios (95% confidence intervals) for the above-mentioned covariates. All statistical analyses were performed using the SPSS statistical package 24 and SAS 9.4.

### Ethics

The study was approved by the Regional Committee for Medical and Health Research Ethics, Region North (REC North, Project No. 2010/2268), and conducted in accordance with the Declaration of Helsinki. To provide accurate data on incidence and prevalence of out-patient commitment, completeness of data was crucial and the REC granted access to medical files without consent by individual patients. All data were de-identified before being stored and used in the analysis.

## Results

### Incidence and prevalence of out-patient commitment

For the whole sample, population-based incidence rates of out-patient commitment varied from 20.7 to 28.4 over the study years (Table 1), while annual point prevalence figures on 31 December each year ranged between 36.5 and 48.9 per 100 000 inhabitants aged 18 years or above in the same period (Table 1). Incidence and prevalence rates varied significantly between sites, from 5.5 to 51.1 and 10.7 to 108.5, respectively (*P* < 0.001). For the whole sample, both incidence and prevalence rates increased over time (test for trend *P* = 0.022 and *P* < 0.001, respectively).

**Table 1:** Prevalence and incidence, *n* and per 100 000 population (total 1700 orders for 1414 people)

	2008	2009	2010	2011	2012
The University Hospital of North Norway					
New out-patient commitment orders, *n*^a^	56	60	57	49	40
Incidence rates^b^	32.3	34.3	32.3	27.5	22.2
Point prevalence, *n*^c^	108	118	130	123	109
Point prevalence rates^b^	62.3	67.5	73.7	68.9	60.4
Innlandet Hospital Trust					
New out-patient commitment orders, *n*^a^	18	17	24	36	33
Incidence rates^b^	5.9	5.5	7.7	11.5	10.4
Point prevalence, *n*^c^	39	33	38	52	55
Point prevalence rates^b^	12.7	10.7	12.2	16.6	17.4
Sørlandet Hospital Trust					
New out-patient commitment orders, *n*^a^	34	28	12	23	19
Incidence rates^b^	42.0	34.1	14.4	27.2	22.2
Point prevalence, *n*^c^	39	54	43	47	46
Point prevalence rates^b^	48.1	65.7	51.7	55.6	53.6
Helse Bergen Trust					
New out-patient commitment orders, *n*^a^	58	95	86	112	92
Incidence rates^b^	19.3	31.0	27.5	35.2	28.4
Point prevalence, *n*^c^	94	125	147	162	153
Point prevalence rates^b^	31.3	40.8	47.0	50.9	47.2
Akershus University Hospital					
New out-patient commitment orders, *n*^a^	40	35	47	60	44
Incidence rates^b^	24.4	21.0	27.8	34.8	25.0
Point prevalence, *n*^c^	60	60	75	90	83
Point prevalence rates^b^	36.6	36.0	44.3	52.2	47.2
Lovisenberg Hospital					
New out-patient commitment orders, *n*^a^	26	32	52	53	56
Incidence rates^b^	27.1	32.0	50.6	50.1	51.1
Point prevalence, *n*^c^	69	72	96	100	119
Point prevalence rates^b^	72.0	72.0	93.5	94.6	108.5
Total					
New out-patient commitment orders, *n*^a^	232	267	278	333	284
Incidence rates^b^	20.7	23.4	24.1	28.4	23.8
Point prevalence, *n*^c^	409	462	529	574	565
Point prevalence rates^b^	36.5	40.6	45.8	48.9	47.4

a. New out-patient commitment orders, *n* = absolute number of orders from 1 Jan to 31 Dec each year.

b. Rates per 100 000 population (18 years of age and above, 1 January each study year).

c. Point prevalence, *n* = absolute number of people at 31 Dec each year.

All first-ever out-patient commitment periods (*n* = 274) took effect at discharge from an involuntary in-patient period. Mean length of hospital stay leading to first-ever out-patient commitment was 124 days (s.d. = 360), with a median of 50 days (Quarter (Q)1–Q3 26–104). Eighteen patients received their first-ever out-patient commitment order directly after their first-ever in-patient period. For the total sample, 10.6% of all compulsory in-patient admissions to the participating sites were converted to out-patient commitment at discharge, varying from 9.2% to 11.9% between sites.

### Duration of out-patient commitment orders

Mean and median duration were 727 (s.d. = 889) and 354 (Q1–Q3 152–980) days respectively (*n* = 1414). Duration of the out-patient commitment episodes differed significantly between sites, from a median of 425 days (Q1–Q3 193–1415) at the University Hospital of North Norway to a median of 273 days (Q1–Q3 118–847) at Innlandet Hospital (*P* = 0.001). At the end of the study period, 429 patients (30.3%) were still under the out-patient commitment order.

For those with the first-ever out-patient commitment episode (*n* = 274), mean duration was 465 days (s.d. = 466) and median duration was 271 days (Q1–Q3 125–713). The mean varied from 370 (s.d. = 423) to 652 (s.d. = 517) days, and the median varied from 170 days (Q1–Q3 99–681) to 525 (Q1–Q3 197–1140) between sites (*P* = 0.065). For 174 (63.5%) patients with a first-ever out-patient commitment, the episode lasted less than a year. Forty (14.6%) patients were still on their first-ever out-patient commitment 3 years after the order was implemented, while five (1.8%) of the patients with a first-time-ever out-patient commitment decision died during the follow-up period.

In the Kaplan–Meier analysis on the 274 patients placed on an out-patient commitment in 2008–2009, the following variables predicted longer duration of out-patient commitment: younger age (*P* = 0.040), having a F20–29 diagnosis (schizophrenia disorders spectrum) (*P* < 0.001), having the first hospital stay more than 3 years ago (*P* = 0.045), living along (*P* = 0.025), having both treatment needs and dangerousness noted as the reasons for an out-patient commitment (*P* = 0.017) and being on depot medication (*P* < 0.001) (Supplementary Fig. 1 available at https://doi.org/10.1192/bjo.2019.60). After mutual adjustment in the Cox regression model, site (*P* = 0.042) also became a significant predictor of duration of out-patient commitment. In addition, longer out-patient commitment duration was predicted by having the first hospital stay more than 3 years ago (*P* = 0.041) and being on depot medication (*P* < 0.001) (Table 2).

**Table 2:** Mutually adjusted hazard ratios for the duration of out-patient commitment order and for the duration between the first-ever out-patient commitment order and time to readmission to a psychiatric facility by background characteristics

Characteristics	Duration of out-patient commitment order (*n* = 242)		Time between the first-ever out-patient commitment and the first readmission to a psychiatric facility (*n* = 241)	
	HR (95% CI)^a^	*P*	HR (95% CI)^a^	*P*
Regional psychiatric hospital				
University Hospital of North Norway	1.23 (0.71 to 2.11)	0.465	2.74 (1.56 to 4.81)	<0.001
Innlandet Hospital Trust	1.83 (0.95 to 3.49)	0.069	2.42 (1.23 to 4.75)	0.010
Sørlandet Hospital Trust	1.97 (1.02 to 3.79)	0.042	1.25 (0.65 to 2.40)	0.501
Helse Bergen Trust	1.42 (0.88 to 2.29)	0.155	2.28 (1.34 to 3.87)	0.002
Lovisenberg Hospital	1.08 (0.63 to 1.86)	0.781	1.00	Ref.
Akershus University Hospital	1.00	Ref.	1.58 (0.87 to 2.89)	0.133
Gender				
Women	1.15 (0.85 to 1.55)	0.370	1.13 (0.82 to 1.56)	0.445
Men	1.00	Ref.	1.00	Ref.
Age				
≥40 years	1.27 (0.91 to 1.79)	0.160	1.25 (0.90 to 1.74)	0.186
18–39 years	1.00	Ref.	1.00	Ref.
Diagnosis				
Affective mood disorders (F31–39)	2.36 (1.52 to 3.66)	<0.001	1.06 (0.70 to 1.62)	0.772
Other	3.16 (1.75 to 5.71)	<0.001	1.31 (0.67 to 2.57)	0.433
Schizophrenia spectrum (F20–29)	1.00	Ref.	1.00	Ref.
First psychiatric hospital stay				
<3 years before out-patient commitment order	1.52 (1.02 to 2.27)	0.041	1.40 (0.88 to 2.18)	0.159
≥3 years before out-patient commitment order	1.00	Ref.	1.00	Ref.
Living conditions				
With someone	1.24 (0.87 to 1.77)	0.236	1.11 (0.77 to 1.60)	0.580
Alone	1.00	Ref.	1.00	Ref.
Involuntary admissions 3 years before out-patient commitment order				
>1 admission	1.03 (0.75 to 1.41)	0.870	1.22 (0.87 to 1.71)	0.244
1 admission	1.00	Ref.	1.00	Ref.
Criteria used for out-patient commitment order:				
Treatment and dangerousness criteria	0.69 (0.46 to 1.05)	0.084	1.10 (0.73 to 1.67)	0.637
Unknown	1.30 (0.62 to 2.72)	0.488	0.64 (0.28 to 1.49)	0.303
Only treatment criteria	1.00	Ref.	1.00	Ref.
Patient taking antipsychotics				
During some of the out-patient commitment period	1.11 (0.73 to 1.68)	0.630	1.53 (0.99 to 2.36)	0.054
During whole out-patient commitment period	1.00	Ref.	1.00	Ref.
Depot injections				
Not receiving	2.14 (1.48 to 3.11)	<0.001	0.78 (0.54 to 1.12)	0.176
Receiving	1.00	Ref.	1.00	Ref.
Involuntary treatment order while on out-patient commitment				
No	0.93 (0.66 to 1.32)	0.696	1.15 (0.80 to 1.63)	0.453
Yes	1.00	Ref.	1.00	Ref.
Psychiatric hospital admissions 3years before out-patient commitment order, *n*	0.94 (0.89 to 1.00)	0.046	1.06 (1.01 to 1.10)	0.014
Days in psychiatric hospital 3 years before out-patient commitment order, *n*	1.00 (1.00 to 1.00)	0.896	1.00 (1.00 to 1.00)	0.827

Ref., reference.

a. Hazard ratios (HRs) are mutually adjusted in multivariable Cox regression analyses. For duration of out-patient commitment orders increased HRs are associated with a shorter duration of out-patient commitment, whereas for time to readmission increased HRs are associated with shorter time to readmissions.

### Compulsory treatment orders

A separate involuntary treatment order was made for 645 (45.6%) of the 1414 patients during their out-patient commitment episode, and the proportion varied by site from 19.3 to 77.5% (*P* < 0.001). Involuntary treatment orders were more prevalent in older patients: 51.4% of those 40 years or older versus 39.1% for those younger than 40 years (*P*<0.001), while there was no gender difference (*P* = 0.078).

### Use of in-patient care 3 years before and after the first-ever out-patient commitment

In total, 209 patients with a first-ever out-patient commitment were studied in this group. Although the number of admissions did not differ significantly between the 3 years before and the 3 years after the first out-patient commitment order (*P* = 0.092), patients had fewer total in-patient days (*P* < 0.001) and fewer in-patient days per admission (*P* = 0.043), in the 3 years after the out-patient commitment order than in the 3 years before (supplementary Table 1). The number of readmissions varied significantly between sites, but increased only at the University Hospital of North Norway (*P* = 0.039). For those who were readmitted, time to first in-patient readmission after first-ever out-patient commitment varied from zero to 1027 days, with a median duration of 55 days (Q1–Q3 5–218). Forty-five patients on their first-ever out-patient commitment were not readmitted during follow-up care.

### Time to readmission

In the Kaplan–Meier analysis (*n* = 257), time between the first-ever out-patient commitment and the first readmission to a mental health facility differed between sites (*P* = 0.006) (Supplementary Fig. 2). All patients used antipsychotic medication, but patients using antipsychotic medication during the whole out-patient commitment period had longer time to readmission than those who took antipsychotic medication but not during the entire period (*P* = 0.008). Further, patients with more than one admission 3 years before the first-ever out-patient commitment were readmitted sooner to a mental health facility (*P* = 0.026). After mutual adjustment in the Cox regression model (*P*<0.001), using antipsychotic medication during the whole out-patient commitment period predicted longer time to readmission (*P* = 0.054), while the number of admissions 3 years before the first-ever out-patient commitment predicted shorter time to readmission (*P* = 0.014) (Table 2).

## Discussion

### Use of out-patient commitment

Incidence and point prevalence rates found in the present study are higher than rates reported from England,^14^ but lower than most jurisdictions in New Zealand and Australia, where rates are reported to be above 100 per 100 000 population at risk.^3,15,16^ This cannot be explained by demographic differences, as our study sample shows the same characteristics as found in most other out-patient commitment studies. However, reservations should be made when incidence and prevalence rates between jurisdictions are compared, because reference populations vary in reports. Some rates are based on total populations, others on adults only and some are based on adults up to the age of 65.^3,14^

Patients were middle aged (30–50 years) with a slight male predominance (56.4%).^4,6,17^ The percentage of all involuntary in-patients who received an out-patient commitment order at discharge varied between 9.2 and 11.9% over the study years, which is about the same level as reported from England,^14^ but lower than other jurisdictions.^18^ The percentages of involuntary in-patients discharged on out-patient commitment orders as well as incidence and prevalence rates differed significantly between sites in the present study. The finding is not surprising as geographical variations within jurisdictions for both in-patient commitment and out-patient commitment are more the rule than the exception.^15,16,19^ The mechanisms underlying such differences are not fully understood, and cannot be explained by demographics, disorder severity or service structures alone.

A general problem related to reports on out-patient commitment incidence and prevalence rates is the varying quality of administrative data. In Norway and Denmark, discrepancies have been found between data recorded by the health authorities and research data based on patients' files. Research data usually identified more cases than those reported by health authorities.^20,21^ A strength of the present study is that data on incidence and prevalence rates are complete, because we were allowed to access all patients' medical files without the consent of individual patients, and data on out-patient commitment in the files were rarely missing. In addition, all data were thoroughly verified by the research team. Thus, we feel confident that the data reported in this study are well suited as a reference point for developments in the use of out-patient commitment, as well as comparisons with other jurisdictions on the use of out-patient commitment.

It is impossible to draw any firm conclusions as to whether a rational use of out-patient commitment is reflected in the proportion of in-patients discharged on an out-patient commitment order, or the incidence and prevalence rates found in this or other studies. To evaluate the use of out-patient commitment orders, one needs to consider the purpose of such orders. Out-patient commitment has different and often ambiguous justifications in different countries. Justifications for the introduction of out-patient commitment include efforts to reduce in-patient care, to increase after-care adherence, to reduce relapse, to prevent revolving-door regimes, to minimise coercive care, to prevent violence or a mixture of these aims. In this context, factors such as the coercive power of out-patient commitment orders, the expressed aims of such orders and how out-patient commitment works in practice, need to be considered. Likewise, the quality and availability of out-patient mental healthcare and social services are important factors when assessing the rationality and efficacy of out-patient commitment regimes.^1^

### Duration of out-patient commitment orders and time to readmission

The median duration of out-patient commitment in our study (354 days) is close to what was found in a study from England that followed up patients on community-treatment orders over 36 months (364 days).^22^ In both studies, the mean duration was longer, reflecting a relatively small group of patients on long periods of out-patient commitment. The determinants of longer out-patient commitment duration found in the current study indicate that patients with a longer history of severe mental illness are likely to spend a longer time under out-patient commitment orders. The differences between sites in duration of out-patient commitment orders may reflect local differences in treatment cultures and service provision. Qualitative interviews with clinicians responsible for patients on an out-patient commitment in Norway revealed different responses to patients who seemed to benefit from the out-patient commitment regime: some clinicians underlined the importance of continuing out-patient commitment for patients who did well, whereas others said they discontinued out-patient commitment because the patients had improved and could be managed without mandated treatment.^23^ The impact of treatment culture and ideology on the use of out-patient commitment is little researched, and future studies on this area should be encouraged.

Our findings that many in-patient episodes before out-patient commitment predicted shorter time to readmission, and that using antipsychotic medication during the whole out-patient commitment period predicted longer time to readmission, could be explained by the same phenomenon, namely adherence to medication, but we are unable to draw any reliable conclusion based on the study data. Interaction between in-patient care, out-patient commitment and voluntary out-patient care is complex, and influential factors might have been omitted from our study. Differences between sites in time to readmission are also likely to reflect local differences in treatment cultures and service provision.

### Outcome of out-patient commitment orders

The most common outcome measure used in assessing out-patient commitment regimes is changes in the use of in-patient services before and after out-patient commitment treatment.^2,9^ In our study we found no reduction in number of admissions, but significant reductions in total number of in-patient days (*P*<0.001) and number of days per admission (*P* = 0.043) when comparing 3 years before with 3 years after implementation of a first-ever out-patient commitment order. The number of readmissions was also reduced, but not significantly (*P* = 0.092). However, all randomised controlled trials on out-patient commitment programmes have found no evidence that out-patient commitment regimes reduce the use of in-patient care.^4,9–11^ In this respect, reservations should be made since the before/after design applied in this study is less robust than randomised controlled designs. Nevertheless, we believe that use of in-patient services as an outcome measure can be questioned regardless of study design. It makes a considerable difference whether the readmission is at the request of the patient or involuntary. Many patients subjected to out-patient commitment have a long psychiatric history and many have poor social supports and networks.^6,17^ Patients may sometimes feel a need for a more protected and safer environment that can be offered by regular out-patient services. Crucial factors in this context are that patients are in control of when they can access and leave in-patient services and that in-patient treatment is on a voluntary basis, despite being under an out-patient commitment order. Such user-initiated in-patient periods may be perceived in a positive light, unlike coercive readmissions to in-patient care. A small-scale study from Norway^24^ on the effect of letting patients with long-term psychotic disorders and comorbid substance misuse decide on in-patient care themselves, is relevant in this context. Despite shortcomings in study design, such as the selection of patients authorised to make self-determined admissions, the outcome was noteworthy: the number of readmissions went up, whereas the length of stay, both per admission and in total, went dramatically down (mean 2.5 days). Most important, involuntary admissions were reduced by 50%.

The question of readmission as an outcome measure in out-patient commitment studies was raised in 2001,^25,26^ but the use of in-patient care is still the most common outcome measure in quantitative studies on out-patient commitment regimes. In the present study, we attempted to record voluntary versus involuntary readmissions, but such data were either lacking or unclear in many cases, which meant that we had to exclude the voluntary/involuntary distinction from the analysis. We are not aware of any outcome studies of out-patient commitment that have distinguished between voluntary and involuntary readmissions. Research incorporating this distinction can provide a better understanding of the impact on in-patient care from a voluntary–involuntary perspective.

### Pattern of out-patient commitment use

All patients in our study with a first-ever out-patient commitment order (*n* = 274) were transferred to out-patient commitment regimes directly after discharge from an in-patient stay. Unfortunately, we did not record whether this applied to the whole sample (*n* = 1414). Our data collectors, however, could not remember any cases of out-patient commitment orders that did not directly follow discharge from an in-patient period. This finding was surprising given the heated public debate when the possibility of making an out-patient commitment order without any prior in-patient period was introduced in 1999. Opponents of the proposal believed that this option represented a widening of the coercive net. Apart from a review from the USA reporting that eight states have the option to place patients on out-patient commitment without any prior in-patient period,^1^ we are not aware of other jurisdictions where direct out-patient commitment placement is a legal option. Why clinicians may refrain from using this possibility needs to be explored in future research.

### Out-patient commitment and involuntary treatment

Just under half of the first-time out-patient commitment patients received an involuntary treatment order, a requirement under the Norwegian Mental Health Act for the treatment of involuntarily placed patients without valid consent. Further, the frequency of involuntary treatment orders varied significantly between sites, from 19.3 to 77.5%. As patients report in qualitative studies, the most controversial issue concerns medication regimes, where patients challenge the kind of medication, dosage, duration and method of drug administration.^12,13^ Why so many patients on an out-patient commitment seem to comply with the medication prescribed without being subject to a separate involuntary treatment order is an interesting question. One possible explanation might be that patients on an out-patient commitment usually have long-term experience of mental health services, including the use of involuntary measures. This has taught them that if they resist taking the prescribed medication, an involuntary treatment order would be made, which could place them in a worse situation than if they comply.^12^ If this holds true, it can be questioned whether patients on an out-patient commitment without an involuntary treatment order take their medication voluntarily based on free consent. Norway is one of the few countries in Europe where a separate order for involuntary treatment is required in addition to the involuntary placement order. The obligation to make separate decisions for involuntary placement and treatment is intended to be an additional legal safeguard for interventions that may represent a serious violation of patients' integrity. The fact that so many patients on an out-patient commitment in this study do not have an involuntary treatment order, and thus miss the opportunity to legally challenge involuntary treatment, is a matter of concern from a patients' rights and patient autonomy perspective.

### Implications

Determining the factors resulting in the pronounced variations in the use of out-patient commitment between sites calls for further study. When considering readmissions as an outcome measure in out-patient commitment studies, it may be important to distinguish between voluntary and involuntary in-patient placement. Also, the reason why only fewer than half of the patients with an out-patient commitment had a separate involuntary treatment order needs to be studied in more detail.

## Supplementary material

For supplementary material accompanying this paper visit http://dx.doi.org/10.1192/bjo.2019.60. click here to view supplementary material

## References

[1] Meldrum ML, Kelly EI, Calderon R, Brekke JS, Braslow JT. Implementation status of assisted outpatient treatment programs: a national survey. Psychiatr Serv 2016; 67: 630–35. 26828396 10.1176/appi.ps.201500073

[2] Barnett P, Matthews H, Lloyd-Evans B, Mackay E, Pilling S, Johnson S. Compulsory community treatment to reduce readmission to hospital and increase engagement with community care in people with mental illness: a systematic review and meta-analysis. Lancet Psychiatry 2018; 5: 1013–22. 30391280 10.1016/S2215-0366(18)30382-1 PMC6251967

[3] O'Donoghue B, Brophy L, Owens N, Rasic M, McCullough B, Huang B, Rate of community treatment orders and readmission orders following reconfiguration of community mental health services. Australas Psychiatry 2016; 24: 278–81. 26849895 10.1177/1039856216629841

[4] Burns T, Rugkåsa J, Molodynski A, Dawson J, Yeeles K, Vazquez-Montes M, Community treatment orders for patients with psychosis (OCTET): a randomised controlled trial. Lancet 2013; 381: 1627–33. 23537605 10.1016/S0140-6736(13)60107-5

[5] World Health Organization. *Mental Health Atlas Country Profile*, 2014 WHO, 2014 ( https://www.who.int/mental_health/evidence/atlas/profiles-2014/nor.pdf).

[6] Rugkåsa J, Nyttingnes O, Simonsen TB, Benth JS, Lau B, Riley H, The use of outpatient commitment in Norway: who are the patients and what does it involve? Int J Law Psychiatry 2019; 62: 7–15. 30616856 10.1016/j.ijlp.2018.11.001

[7] Sjøstrøm S, Zetterberg L, Markstrom U. Why community compulsion became the solution — reforming mental health law in Sweden. Int J Law Psychiatry 2011; 34: 419–28. 22104265 10.1016/j.ijlp.2011.10.007

[8] Lawton-Smith S, Dawson J, Burns T. Community treatment orders are not a good thing. Br J Psychiatry 2008; 193: 96–100. 18669989 10.1192/bjp.bp.107.049072

[9] Kisely S, Hall K. An updated meta-analysis of randomized controlled evidence for the effectiveness of community treatment orders. Can J Psychiatry 2014; 59: 561–4. 25565690 10.1177/070674371405901010 PMC4197791

[10] Steadman HJ, Gounis K, Dennis D, Hopper K, Roche B, Swartz M, Assessing the New York City involuntary outpatient commitment pilot program. Psychiatr Serv 2001; 52: 330–6. 11239100 10.1176/appi.ps.52.3.330

[11] Swartz M, Swanson JD, Wagner H, Burns B, Hiday V, Borum R. Can involuntary outpatient commitment reduce hospital recidivism? Findings from a randomized trial with severely mentally ill individuals. Am J Psychiatry 1999; 156: 1968–75. 10588412 10.1176/ajp.156.12.1968

[12] Riley H, Høyer G, Lorem GF. ‘When coercion moves into your home’ - a qualitative study of patient experiences with outpatient commitment in Norway. Health Soc Care Comm 2014; 22: 506–14. 10.1111/hsc.12107 24703340

[13] Corring D, O'Reilly R, Sommerdyck C. A systematic review of the views and experiences of subjects of community treatment orders. Int J Law Psychiatry 2017; 52: 74–80. 28325533 10.1016/j.ijlp.2017.03.002

[14] Trevithick L, Carlile J, Nodiyal S, Keown P. Community treatment orders: an analysis of the first five years of use in England. Br J Psychiatry 2018; 212: 175–9. 29439748 10.1192/bjp.2017.51

[15] O'Brien AJ. Community treatment orders in New Zealand: regional variability and international comparison. Australas Psychiatry 2014; 22: 352–6. 24733307 10.1177/1039856214531080

[16] Light E, Kerridge I, Ryan C, Robertson M. Community treatment orders in Australia: rates and pattern of use. Australas Psychiatry 2012; 20: 478–82. 23136187 10.1177/1039856212466159

[17] Churchill R, Owen G, Singh S, Hotopf M. International Experiences of Using Community Treatment Orders. Department of Health, 2007.

[18] Burgess P, Bindman J, Leese M, Henderson C, Szmukler G. Do community treatment orders for mental illness reduce readmission to hospital? Soc Psychiatry Psychiatr Epidemiol 2006; 41: 574–9. 16685479 10.1007/s00127-006-0063-1

[19] Weich SR, McBride O, Twigg L, Duncan C, Keown P, Crepaz-Keay D, Variation in compulsory psychiatric inpatient admission in England: a cross-classified multilevel analysis. Lancet Psychiatry 2017; 4: 619–26. 28647537 10.1016/S2215-0366(17)30207-9

[20] Iversen K, Høyer G, Sexton H. Rates of civil commitment to psychiatric institutions in Norway. Can national statistics be trusted? Nord J Psychiatry 2009; 63: 301–7. 19199121 10.1080/08039480902730607

[21] Poulsen HD. The prevalence of extralegal deprivation of liberty in a psychiatric hospital population. Int J Law Psychiatry 2002; 25: 29–36. 12089777 10.1016/s0160-2527(01)00093-0

[22] Burns T, Yeeles K, Koshiaris C, Vazquez-Montes M, Molodynski A, Puntis S, Effect of increased compulsion on readmission to hospital or disengagement from community services for patients with psychosis: follow-up of a cohort from the OCTET trial. Lancet Psychiatry 2015; 10: 881–90. 10.1016/S2215-0366(15)00231-X 26362496

[23] Riley H, Lorem GF, Høyer G. Community treatment orders - what are the views of decision makers? J Ment Health 2016; 2: 97–102. 10.1080/09638237.2016.1207230 27461530

[24] Heskestad S, Tytlandsvik M. Brukerstyrte kriseinnleggelser ved alvorlig psykisk lidelse [User-driven emergency admissions for serious mental illness]. Tidsskr Nor Legeforen 2008; 128: 32–5. 18183054

[25] Høyer G, Ferris R. Outpatient commitment. Some reflections on ideology, practice and implications for research. J Mental Health Law 2001; 6: 56–65.

[26] Draine J. Conceptualizing service research on outpatient commitment. J Mental Health Admin 1997; 24: 306–15. 10.1007/BF02832664 9230572

