# Supplementary material for: Out-patient commitment order use in Norway: incidence and prevalence rates, duration and use of mental health services from the Norwegian Outpatient Commitment Study
Source: BJPsych Open. 2019 Sep 2;5(5):e75. doi: 10.1192/bjo.2019.60 (PMC6737513; doi:10.1192/bjo.2019.60)
Supplement: Supplementary file 1 [file bjosup.zip › S2056472419000607sup002.docx]

Supplementary Table 1 Use of in-patient care for patients on their first ever OC order, three years before and three years after (*n* = 209)

|  | Three years before index OC order^a^ | Three years after index OC order | *P* value^b^ |
| --- | --- | --- | --- |
| The University Hospital of North Norway | 38 | 38 |  |
| Number of inpatient admissions |  |  | 0.039 |
| Mean (s.d.) | 3 (3) | 6 (7) |  |
| Range | 1–14 | 0–34 |  |
| Median (Q1–Q3) | 2 (1; 3) | 3 (1; 8) |  |
| Cumulative days, *n* |  |  | 0.324 |
| Mean (s.d.) | 77 (58) | 110 (147) |  |
| Range | 7–278 | 0–717 |  |
| Median (Q1–Q3) | 62 (31; 106) | 61 (10; 138) |  |
| Mean number of days per admission |  | *n* = 31 | 0.167 |
| Mean (s.d.) | 34 (26) | 23 (25) |  |
| Range | 7–120 | 5–138 |  |
| Median (Q1–Q3) | 25 (18; 46) | 17 (10; 26) |  |
| Innlandet Hospital Trust | 21 | 21 |  |
| Number of inpatient admissions |  |  | 0.160 |
| Mean (s.d.) | 4 (3) | 3 (3) |  |
| Range | 1–10 | 0–13 |  |
| Median (Q1–Q3) | 4 (2; 5) | 3 (2; 4) |  |
| Cumulative days, *n* |  |  | 0.017 |
| Mean (s.d.) | 244 (192) | 118 (130) |  |
| Range | 37–733 | 0–426 |  |
| Median (Q1–Q3) | 190 (134; 278) | 56 (14; 191) |  |
| Mean number of days per admission |  | *n* = 18 | 0.122 |
| Mean (s.d.) | 96 (126) | 40 (50) |  |
| Range | 11–573 | 2–213 |  |
| Median (Q1–Q3) | 39 (29; 151) | 26 (13; 36) |  |
| Sørlandet Hospital Trust | 24 | 24 |  |
| Number of inpatient admissions |  |  | 0.109 |
| Mean (s.d.) | 6 (5) | 5 (6) |  |
| Range | 1–20 | 0–19 |  |
| Median (Q1–Q3) | 4 (3; 8) | 2 (1; 6) |  |
| Cumulative days, *n* |  |  | 0.011 |
| Mean (s.d.) | 137 (113) | 79 (159) |  |
| Range | 7–408 | 0–774 |  |
| Median (Q1–Q3) | 102 (48; 220) | 27 (4; 78) |  |
| Mean number of days per admission |  | *n* = 18 | 0.058 |
| Mean (s.d.) | 29 (22) | 17 (17) |  |
| Range | 7–82 | 3–74 |  |
| Median (Q1–Q3) | 25 (13; 32) | 12 (6; 23) |  |
| Helse Bergen Trust | 74 | 74 |  |
| Number of inpatient admissions |  |  | 0.284 |
| Mean (s.d.) | 3 (3) | 3 (3) |  |
| Range | 1–20 | 0–15 |  |
| Median (Q1–Q3) | 2 (1; 3) | 2 (1; 4) |  |
| Cumulative days, *n* |  |  | 0.028 |
| Mean (s.d.) | 205 (274) | 159 (256) |  |
| Range | 6–1096 | 0–1095 |  |
| Median (Q1–Q3) | 111 (48; 232) | 62 (10; 173) |  |
| Mean number of days per admission |  | *n* = 60 | 0.735 |
| Mean (s.d.) | 106 (213) | 96 (201) |  |
| Range | 2–1096 | 1–1095 |  |
| Median (Q1–Q3) | 42 (27; 76) | 31 (16; 72) |  |
| Akershus University Hospital | 26 | 26 |  |
| Number of inpatient admissions |  |  | 0.678 |
| Mean (s.d.) | 2 (2) | 2 (2) |  |
| Range | 1–6 | 0–9 |  |
| Median (Q1–Q3) | 2 (1; 3) | 2 (0; 3) |  |
| Cumulative days, *n* |  |  | 0.213 |
| Mean (s.d.) | 171 (193) | 116 (142) |  |
| Range | 6–878 | 0–423 |  |
| Median (Q1–Q3) | 114 (40; 212) | 68 (0; 153) |  |
| Mean number of days per admission |  | *n* = 18 | 0.845 |
| Mean (s.d.) | 89 (109) | 58 (49) |  |
| Range | 6–518 | 5–189 |  |
| Median (Q1–Q3) | 55 (20; 118) | 46 (25; 71) |  |
| Lovisenberg Hospital | 26 | 26 |  |
| Number of inpatient admissions |  |  | 0.013 |
| Mean (s.d.) | 5 (6) | 2 (3) |  |
| Range | 1–22 | 0–9 |  |
| Median (Q1–Q3) | 3 (2; 6) | 1 (1; 3) |  |
| Cumulative days, *n* |  |  | 0.001 |
| Mean (s.d.) | 182 (173) | 66 (71) |  |
| Range | 5–888 | 0–240 |  |
| Median (Q1–Q3) | 153 (69; 257) | 42 (6; 120) |  |
| Mean number of days per admission |  | *n* = 19 | 0.494 |
| Mean (s.d.) | 83 (170) | 45 (41) |  |
| Range | 3–888 | 3–127 |  |
| Median (Q1–Q3) | 45 (23; 66) | 26 (10; 71) |  |
| Total | 209 | 209 |  |
| Number of inpatient admissions |  |  | 0.092 |
| Mean (s.d.) | 4 (4) | 3 (5) |  |
| Range | 1–22 | 0–34 |  |
| Median (Q1–Q3) | 2 (1; 4) | 2 (1; 4) |  |
| Cumulative days, *n* |  |  | <0.001 |
| Mean (s.d.) | 171 (206) | 120 (188) |  |
| Range | 5–1096 | 0–1095 |  |
| Median (Q1–Q3) | 114 (50; 202) | 57 (8; 141) |  |
| Mean number of days per admission^c^ |  | *n* = 164 | 0.043 |
| Mean (s.d.) | 78 (153) | 57 (128) |  |
| Range | 2–1096 | 1–1095 |  |
| Median (Q1–Q3) | 36 (21; 69) | 23 (11; 57) |  |

OC, outpatient commitment; SD, standard deviation; Q1–Q3, first and third quartiles.

a. 51 patients with fewer than three years’ observation time and 14 people were excluded because of poor quality of the data (*n* = 209).

b. Wilcoxon Signed Ranks test was used to test for the differences before and after.

c. 45 people had no admissions three years after their first ever CO order.
