# Supplementary material for: Out-patient commitment order use in Norway: incidence and prevalence rates, duration and use of mental health services from the Norwegian Outpatient Commitment Study
Source: BJPsych Open. 2019 Sep 2;5(5):e75. doi: 10.1192/bjo.2019.60 (PMC6737513; doi:10.1192/bjo.2019.60)
Supplement: Supplementary file 1 [file bjosup.zip › S2056472419000607sup001.docx]

**Figure 1.** A panel with Kaplan-Meier survival plots for the duration of outpatient commitment (OC) by different levels of potential predictors.


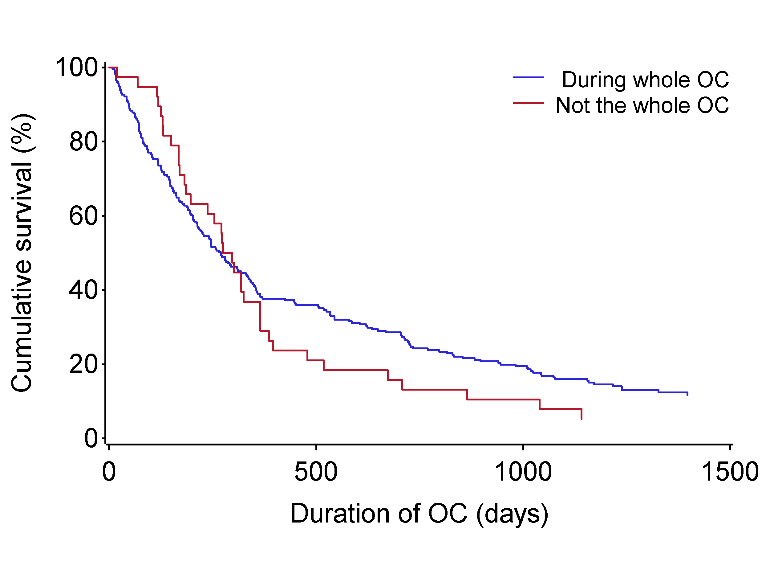

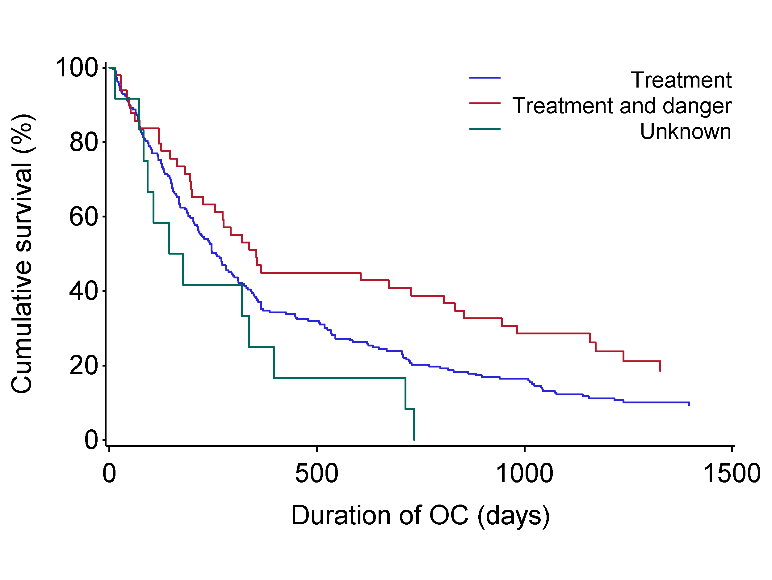

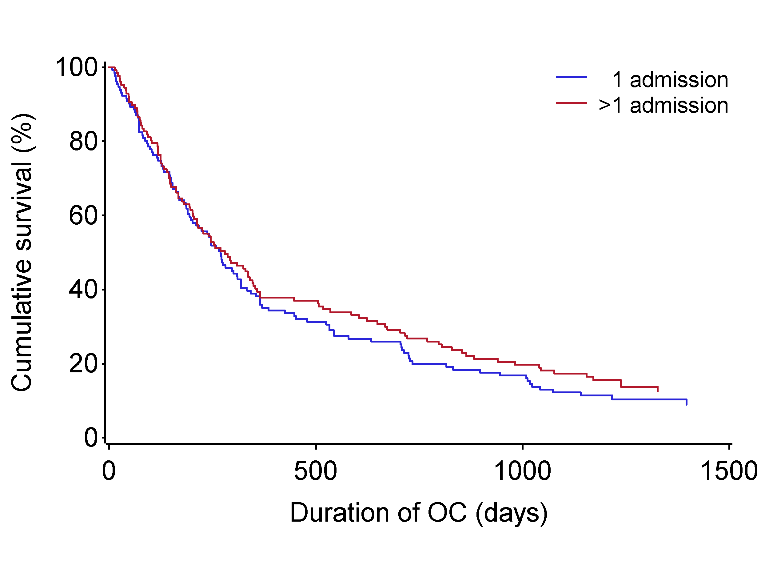

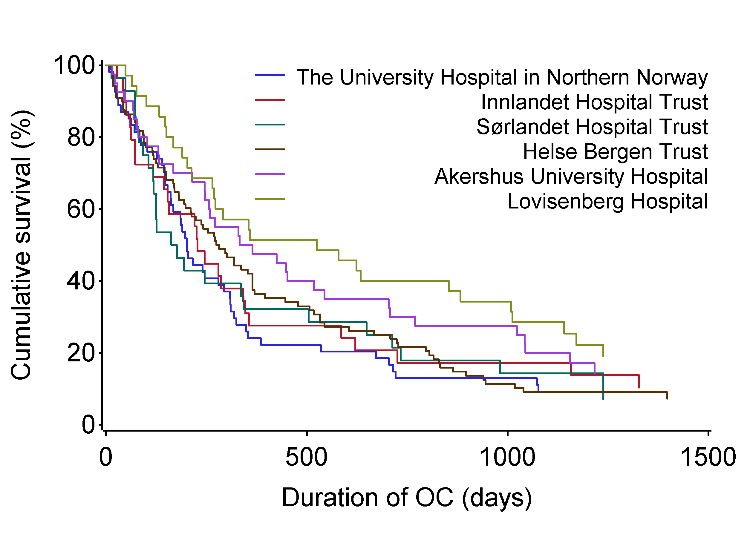
  **
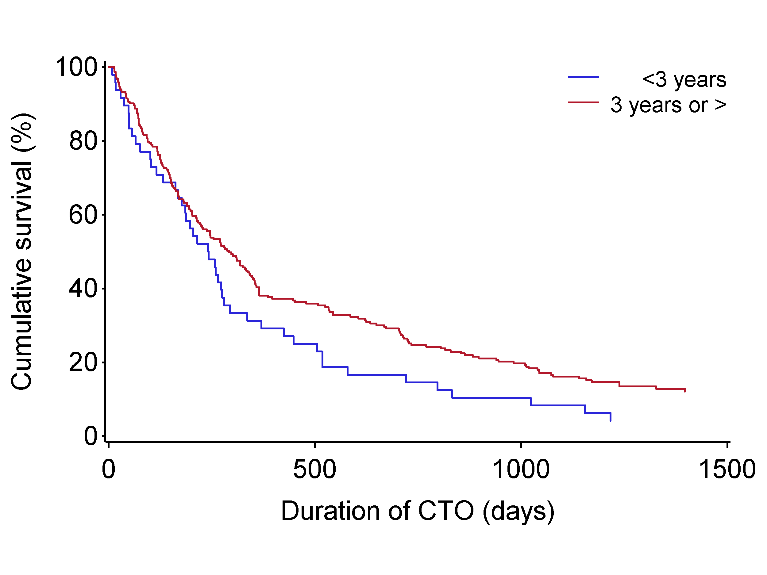

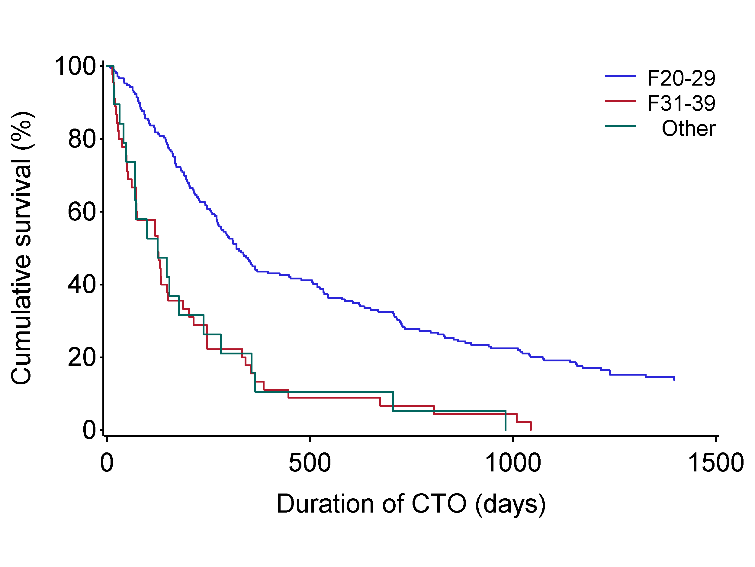

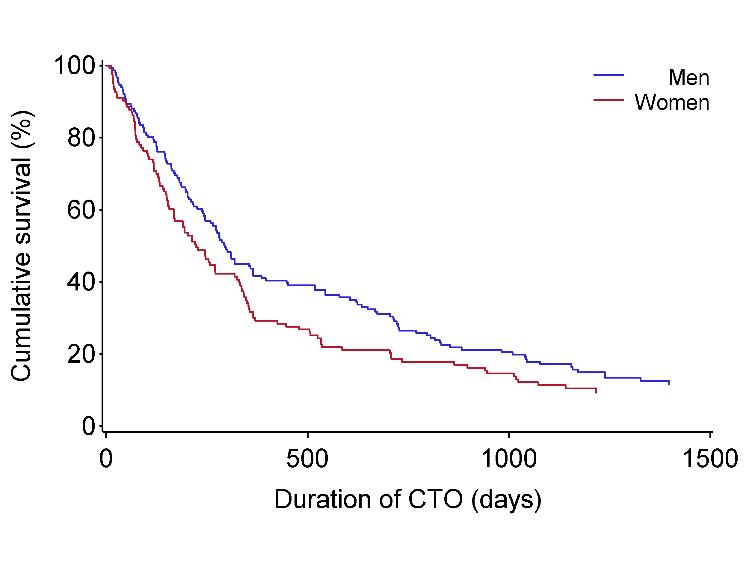

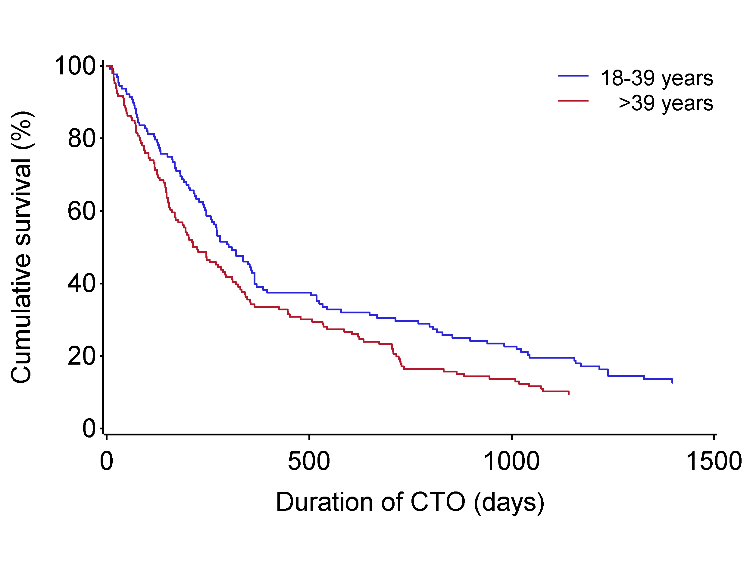
**

**p=0.373**

**p=0.017**

**Criteria used for OC**

**p=0.365**

**Involuntary admissions**

**before OC**

**p=0.025**

**Living conditions**

**First psychiatric hospital stay**

**p=0.045**

**p<0.001**

**Diagnosis**

**Age**

**p=0.040**

**p=0.117**

**p=0.061**

**Gender**


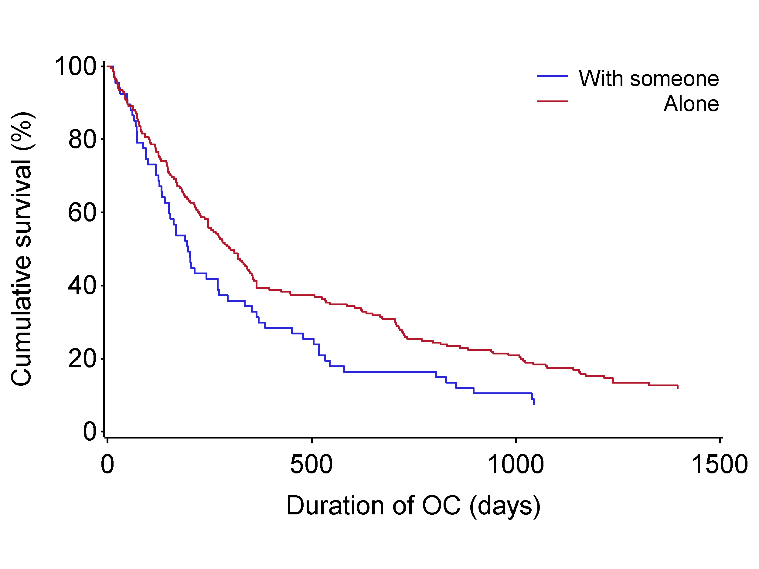


**Continued**

**Patient on medication**

**Figure 1.** A panel with Kaplan-Meier survival plots for the duration of outpatient commitment (OC) by different levels of potential predictors.


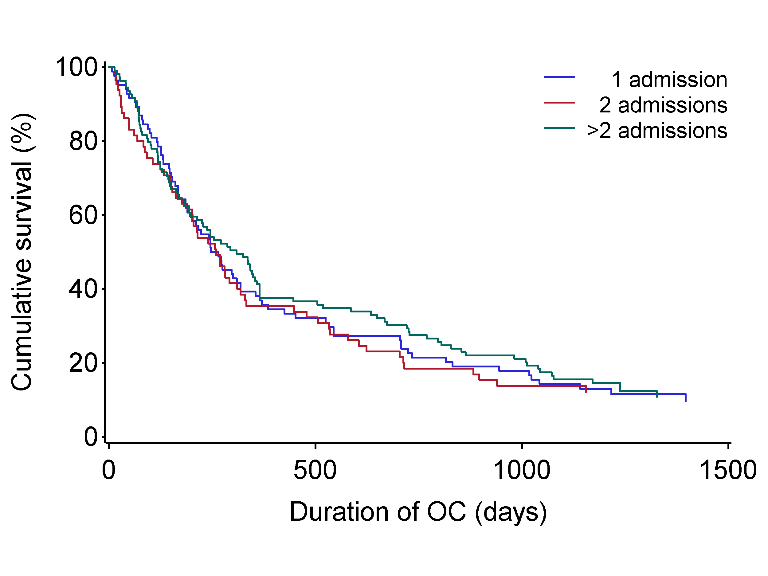

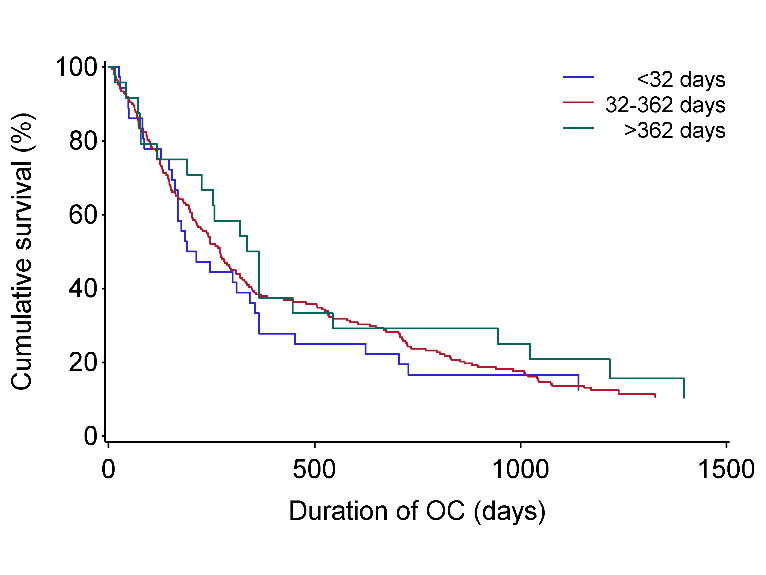

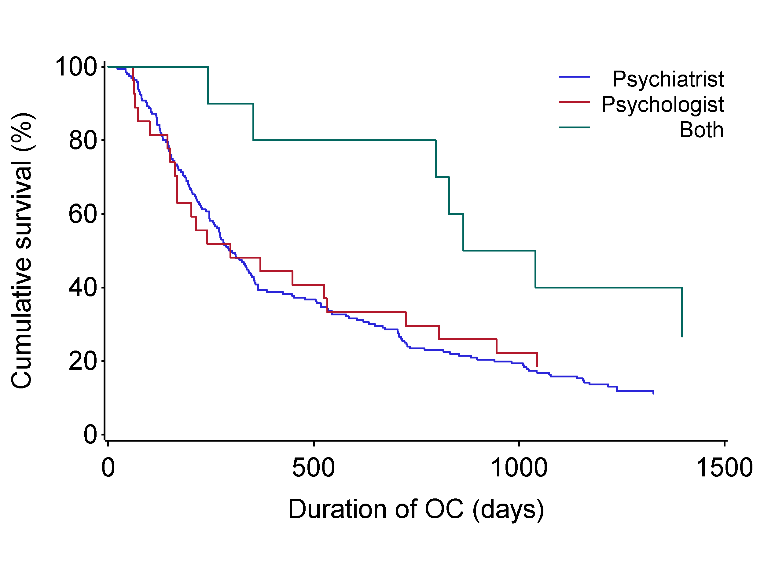

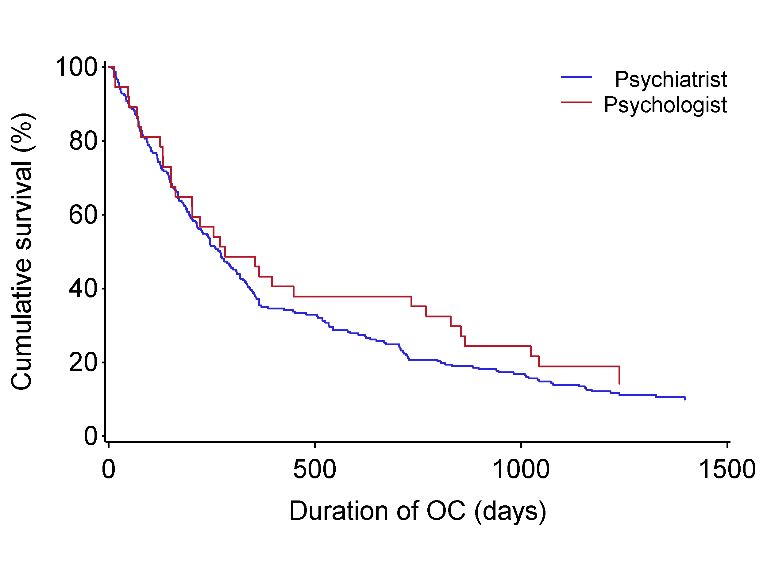
 **
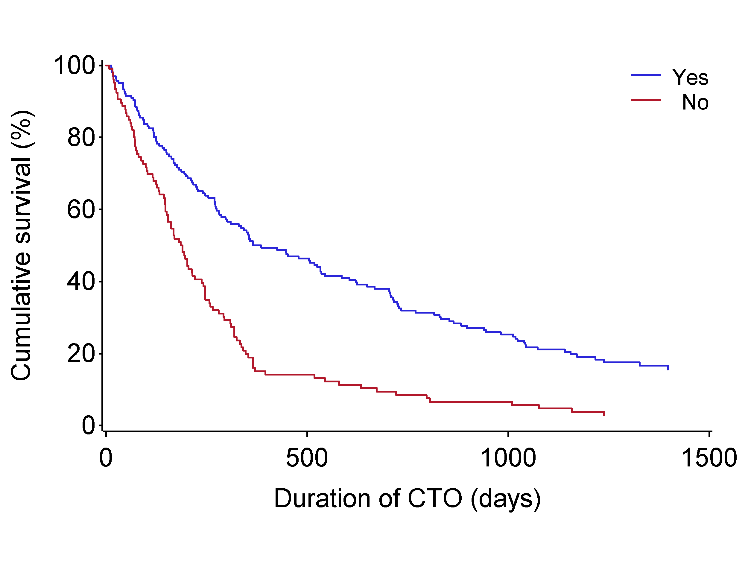

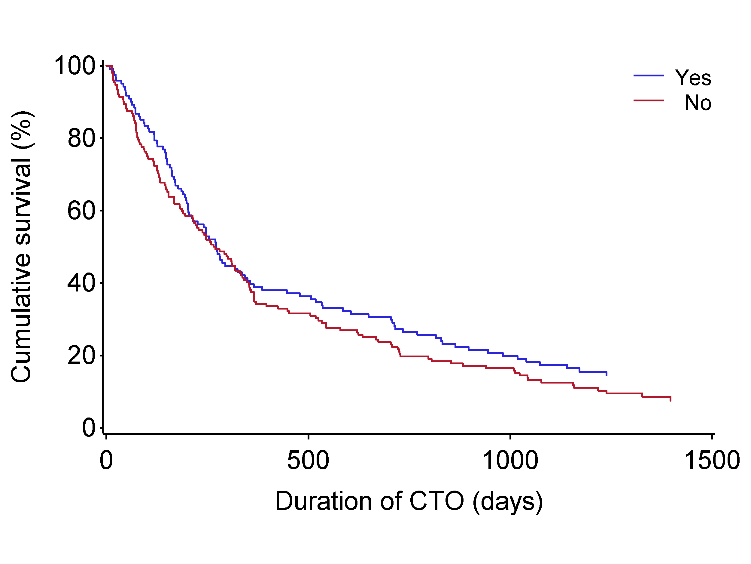
Continued**

**p=0.753**

**Number of admissions**

**before OC**

**Days in psychiatric hospital 3 years before**

**p=0.748**

**p=0.080**

**Formal responsibility**

**p<0.001**

**Depot injections**

**p=0.181**

**p=0.309**

**Responsible for OC order**

**Involuntary treatment order while on OC**

**Figure 2.** A panel with Kaplan-Meier survival plots for the duration between the first ever outpatient commitment (OC) and the first readmission to a psychiatric facility by different levels of potential predictors.

**
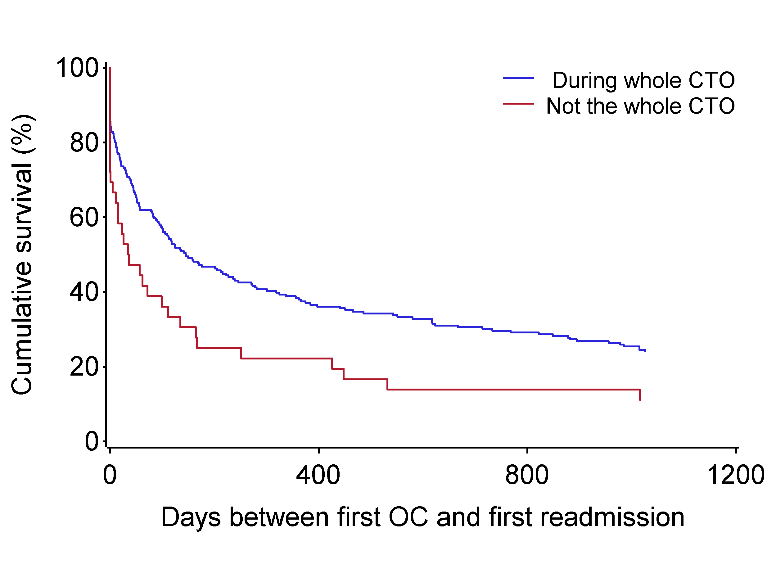

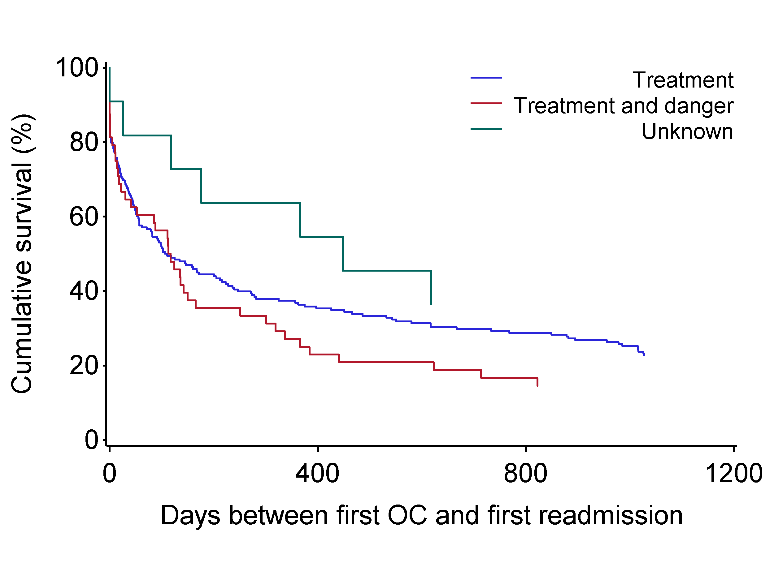

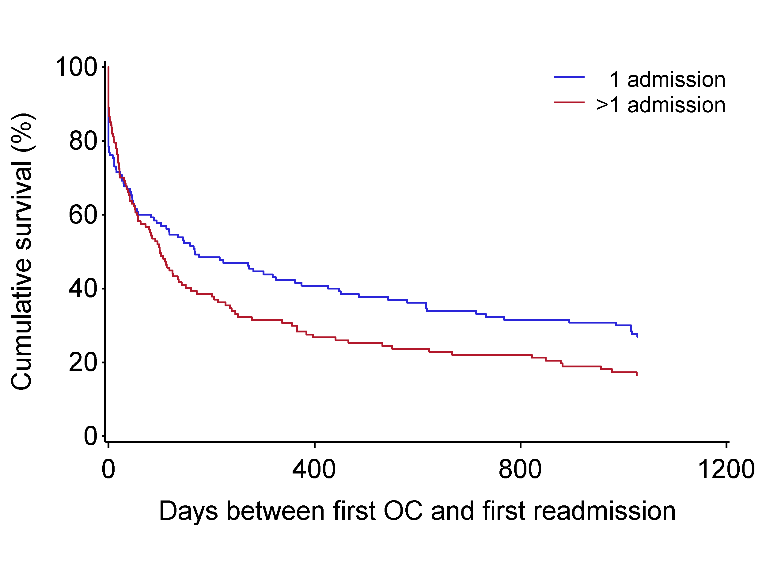

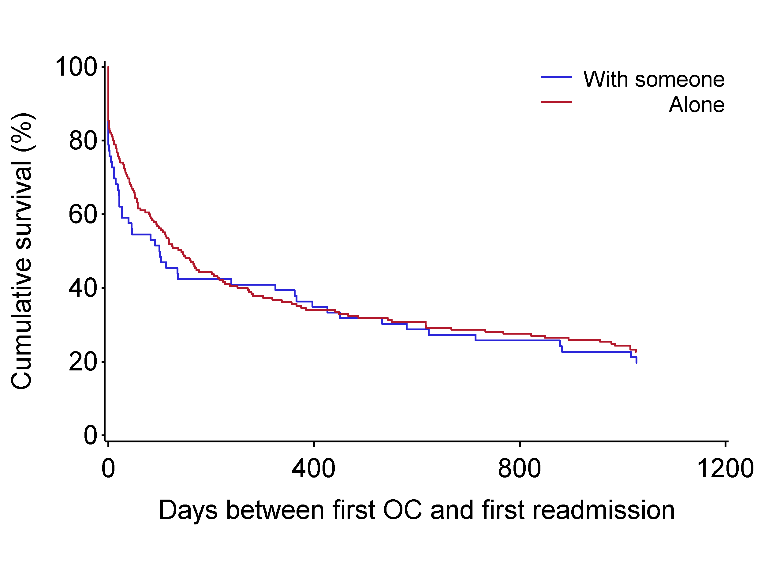

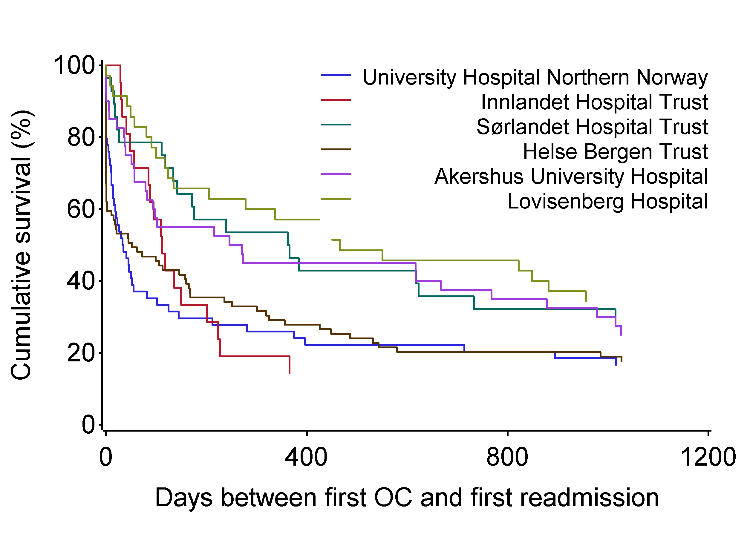

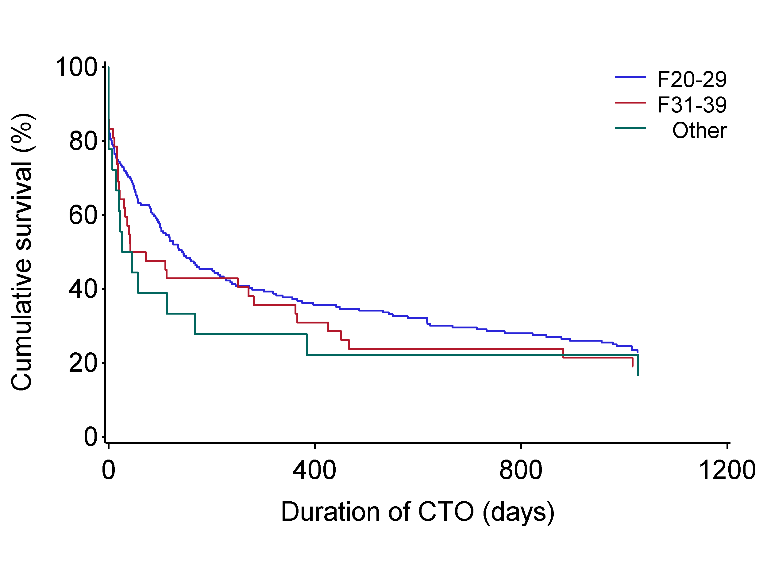

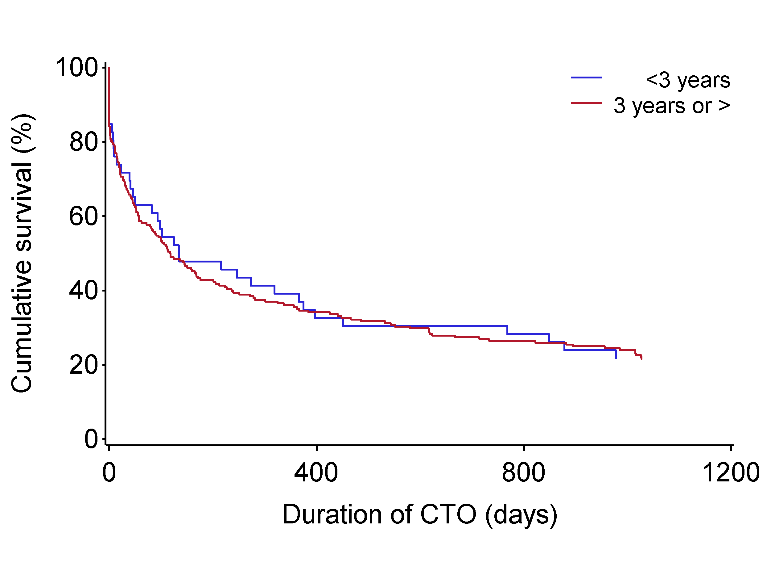

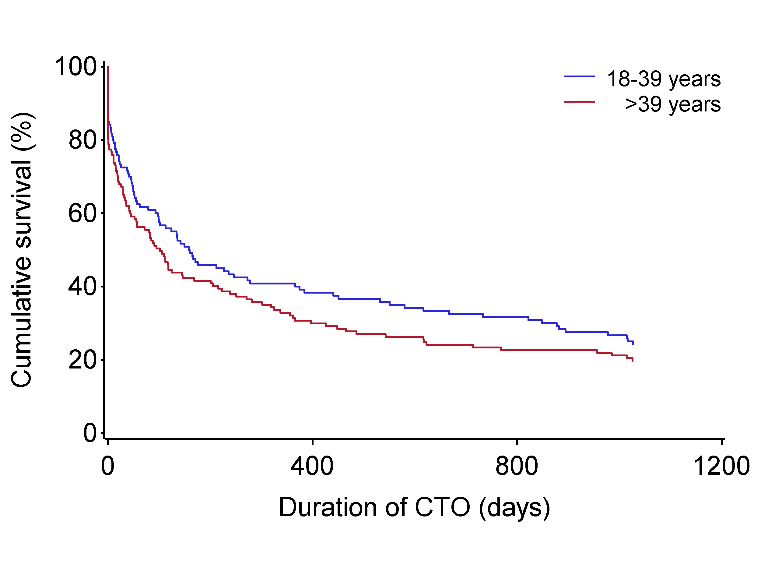

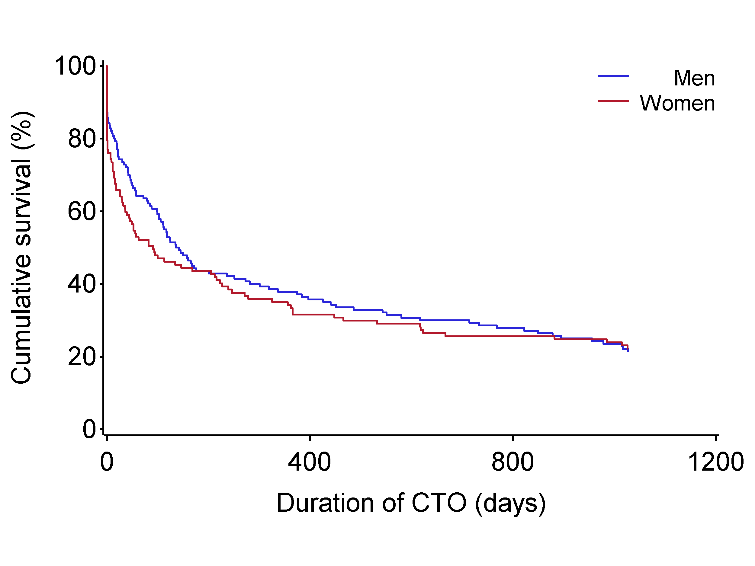
**

**p=0.008**

**Patient on medication**

**Criteria used for OC**

**p=0.197**

**Involuntary admissions**

**before OC**

**p=0.057**

**p=0.488**

**Living conditions**

**First psychiatric hospital stay**

**Diagnosis**

**p=0.854**

**p=0.390**

**p=0.196**

**p=0.539**

**p=0.006**

**Gender**

**Age**

**Continued**

**Figure 2.** A panel with Kaplan-Meier survival plots for the duration between the first ever outpatient commitment (OC) and the first readmission to a psychiatric facility by different levels of potential predictors.

**
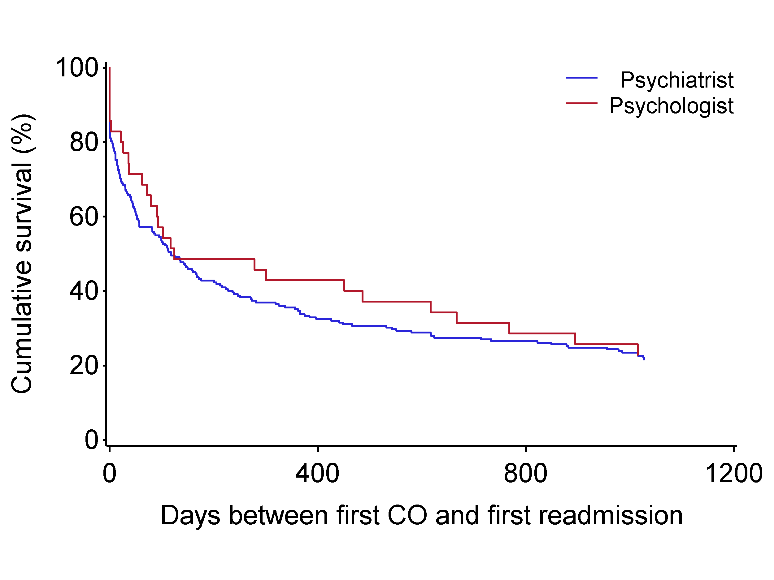

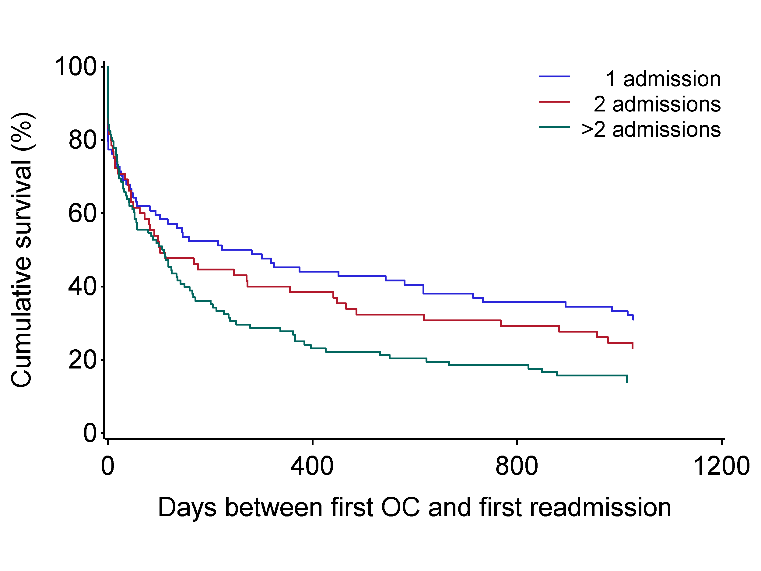

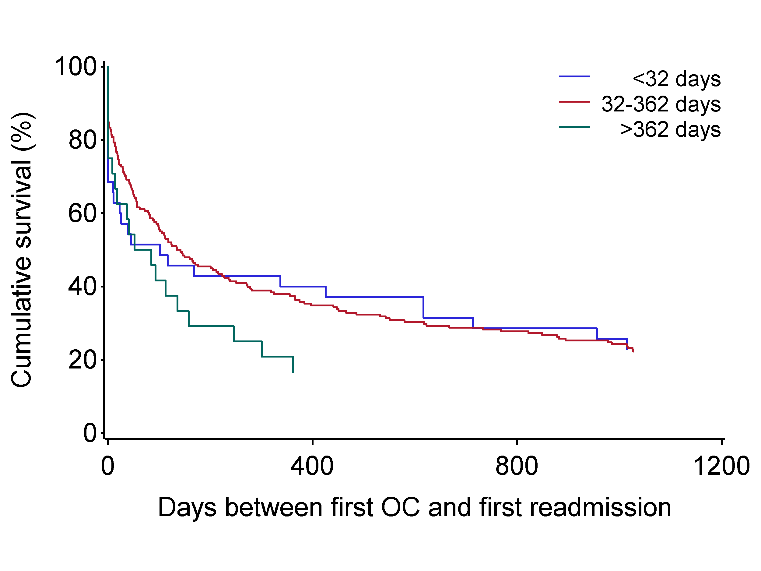

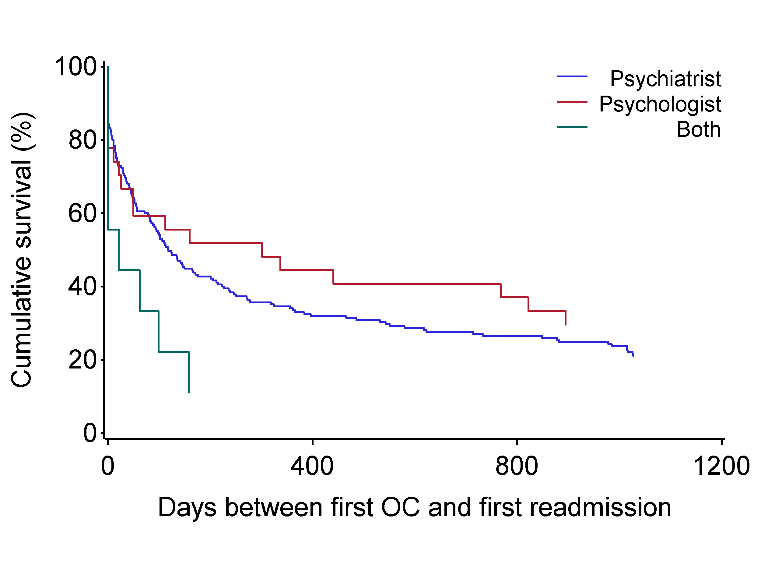

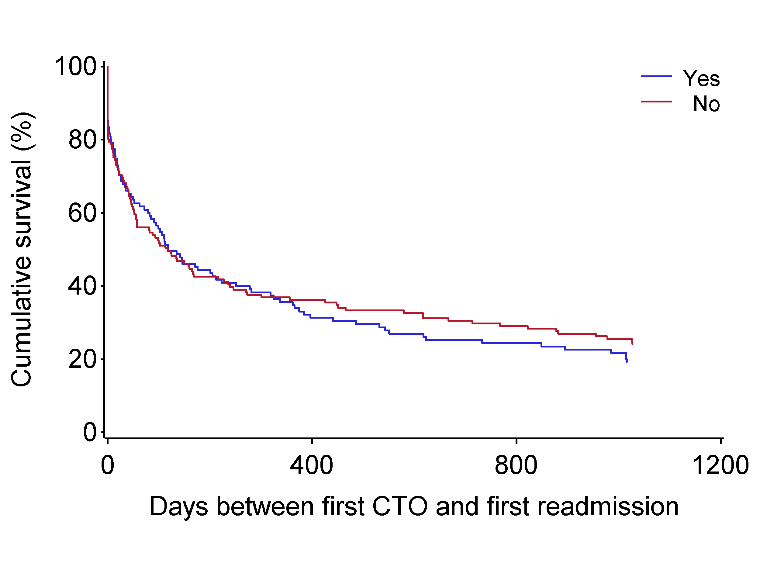

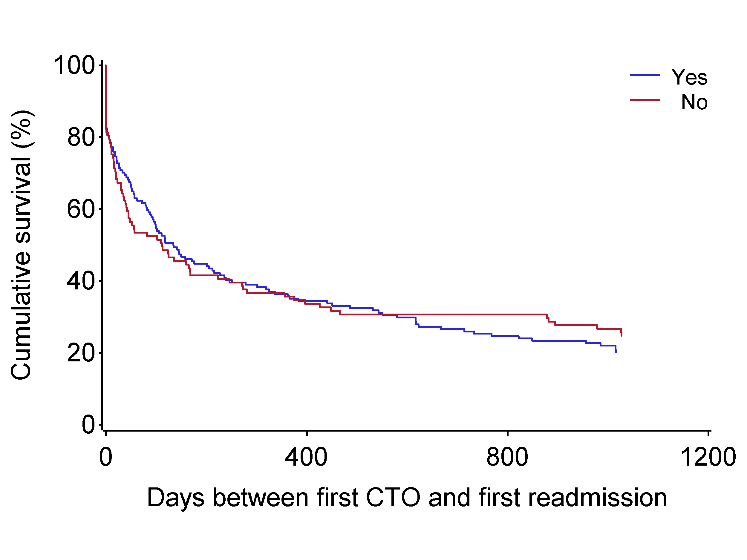
Continued**

**Number of admissions**

**before OC**

**Days in psychiatric hospital 3 years before**

**Formal responsibility**

**p=0.026**

**p=0.127**

**p=0.410**

**Depot injections**

**Involuntary treatment order while on OC**

**p=0.597**

**p=0.803**

**Responsible for OC order**

**p=0.565**
